# Dosimetric perturbation from cloth and paper gowns for total skin electron irradiation

**DOI:** 10.1120/jacmp.v14i4.4045

**Published:** 2013-07-08

**Authors:** James P. Steinman, Shane L. Hopkins, Iris Z. Wang

**Affiliations:** ^1^ Department of Radiation Medicine Roswell Park Cancer Institute Buffalo NY; ^2^ Department of Physiology and Biophysics State University of New York at Buffalo Buffalo NY; ^3^ Department of Radiation Oncology William R Bliss Cancer Center, Mary Greely Medical Center Ames IA USA

**Keywords:** total skin electron irradiation, dose, clothing, gown

## Abstract

Traditionally, total skin electron patients remove all clothing for treatment. It is generally assumed that this is best for the treatment of superficial skin lesions out of concern clothing may significantly perturb dose. We investigate the dosimetric effect of patient gowns and determine the necessity of treating patients naked. Using GAFCHROMIC EBT2 film, dose to a cylindrical phantom was measured with cloth, paper, and tri‐layer cloth gowns, compared to no covering. A 6 MeV electron beam with spoiler accessory was used at ∼4 meters source‐to‐skin distance. The gantry was angled at 248° and 292°. The phantom was rotated at ‐60°, 0°, and 60° relative to the beam's central axis, simulating the Stanford technique. This was also repeated for films sandwiched between the phantom's discs. Using a Markus chamber, the effect of air gaps of 0 to 5 cm in cloth and paper gowns was measured. The water‐equivalent attenuation of the gowns was determined through transmission studies. Compared to no covering, films placed on the phantom surface revealed an average increase of 0.8% in dose for cloth, 1.8% for tri‐layered cloth, and 0.7% for paper. Films sandwiched within the phantom showed only slight shift of the percent depth‐dose curves. Markus chamber readings revealed 1.4% for tri‐layered cloth, and <0.2% for single layer cloth or paper. Air gaps appeared to have a minimal effect. Transmission measurements found that one layer of cloth is equal to 0.2 mm of solid water. Cloth and paper gowns appear to slightly increase the dose to the skin, but will not introduce any significant dose perturbation (<1%). Gowns having folds and extra layers will have a small additional perturbation (<2%). To minimize perturbation, one should smooth out any folds or remove any pockets that form extra layers on the gown.

PACS number: 87.53.Bn

## I. INTRODUCTION

Total skin electron irradiation (TSEI) is used for the treatment of mycosis fungoides and other types of cutaneous lymphoma lesions.^(1)^ The common practice of TSEI involves 6 MeV electrons attenuated through a Lucite spoiler at a high repetition rate (e.g., 888 MU/min with Varian Clinac series linear accelerators). The positioning of the patient and the linear accelerator gantry is followed according to the Stanford technique to adequately and uniformly irradiate the patient's skin. During these treatments, patients are treated naked due to the concern that the effect of even a thin covering of clothing, such as a patient gown, would cause a significant dose perturbation. Published data regarding clothing on TSEI patients are scarce. However, using TLD measurements, a previous poster presentation^(2)^ concluded that cloth gowns do not significantly alter electron skin dose. Using radiochromic film and ion chamber measurements, we seek to add to the published data regarding this clinical issue and determine what potential dosimetric risks there are for patients who remain gowned during treatment.

Accurate dosimetry of TSEI beams may be challenging, particularly due to the shallow depth and sharp gradients of the low‐energy electrons utilized. The treatment depth for TSEI is on the order of several millimeters, which makes certain dosimeters unsuitable as the point of measurement is beyond this thickness. In the case of ion chambers, for low‐energy electron beams it is necessary to use plane‐parallel chambers which measure much closer to their surface, as opposed to thicker cylindrical chambers used for high‐energy electron beams.^(3)^ For silicon diodes, these are not accurate dosimeters for shallow depths, but may be used for relative measurements if the beam has been independently verified through ion chamber measurements.^(4)^ Film has been well‐known as an accurate dosimeter for electron beams. Radiochromic film, in particular, has been shown to be useful for electrons as it is effectively independent of energy and dose rate^(5)^ and has been used in studies of TSEI providing accurate results.^6^, ^7^ Other effective dosimeters for TSEI include thermoluminescent dosimeters (TLD), gel dosimetry, or other devices that can measure sharp gradients and shallow depth. For our measurements, we specifically used the Markus chamber and radiochromic film.

## II. MATERIALS AND METHODS

### A. Film setup

#### A.1 Surface dose measurement

For our measurements, we cut out small sections of a cloth gown made from cotton. Since these gowns often have pockets or may have folds, we made a section that had three layers of cloth for comparison. We also cut out sections of a paper gown, which is much lighter than the cloth gowns but not used as regularly on patients.

**Figure 1:**
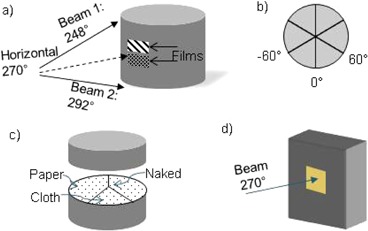
Experimental setups used: (a) surface dose measurement setup of cylindrical phantom with two EBT2 films on surface; (b) cylindrical phantom from above showing 0 and ±60° marks used for the Stanford technique; (c) depth profile measurement setup of GAFCHROMIC film between discs of cylindrical phantom indicating which covering option was placed on the surface of each sector; (d) Markus chamber setup of Lucite phantom.

Two GAFCHROMIC EBT2 films (International Specialty Products ISP, Wayne, NJ) were placed on a cylindrical phantom near the central axis of a horizontal 270° beam (Fig. 1(a)). The total skin dose near the center is highly uniform as verified by our commissioning and annual QA data; even though the films were about 1 cm apart vertically, this will not significantly affect the dose distribution. One film was covered by a strip of cloth, tri‐layered cloth, or paper gown while the other, for control purposes, was left uncovered. The purpose of each material having its own control film is to accommodate for any uncertainty introduced by setup and positional error. In accordance with the six‐field Stanford technique, the orientation of the phantom was rotated from 0° to ‐60° to 60° (Fig. 1(b)) each for gantry angles of 248° and 292°.(^3^) We used a Varian Clinac 2100 C/D (Varian Medical Systems, Palo Alto, CA) in total skin electron mode with 6 MeV electrons and ∼1cm Lucite spoiler accessory.

For dosimetric analysis, an H&D characteristic curve for the films was created using 6 MeV electrons. All the films were scanned using a Vidar VXR‐16 scanner (Vidar Systems Corporation, Herndon, VA) and analyzed using RIT113 Version 5.1 (Radiological Imaging Technology, Colorado Springs, CO). For each material, the average dose of the film was obtained and compared with its corresponding control film for the percent difference.

With GAFCHROMIC EBT2, as suggested by the vendor, the orientation of the film as the dose response will vary, whether the film is in the portrait or landscape position when shot and scanned. To prevent any such errors, all EBT2 films were marked at a corner to indicate its orientation. Also, as recommended by the manufacturer, the films were scanned at least 24 hours after exposure to allow adequate time for the chemical reaction induced by the ionizing radiation to be completed. In addition, all films were taken from the same batch, including those used for the H&D curve. Despite taking precautions, it should be noted that there still appeared significant noise within the measurements; a median filter within the RIT software helped smooth out the dose profiles for our measurements.

#### A.2 Depth profile measurement

A sheet of the GAFCHROMIC EBT2 film was cut to fit the circular shape of the cylindrical phantom and sandwiched between two of the discs (Fig. 1(c)). A strip of a cloth gown and a paper gown were placed on different parts of the phantom surface. The phantom was then irradiated according to the six‐field Stanford technique. The films were scanned and the difference in percentage depth dose (Pdd) profiles was analyzed.

### B. Markus chamber measurement setup

The Markus chamber used was a PTW‐Freiburg N 23343–1515 (PTW, Freiburg, Germany) with a 1 mm water‐equivalent buildup cap. Readings were read using a Keithley 35617 electrometer (Keithley Instruments, Cleveland, OH). The chamber was embedded in a specially made 5 cm thick Lucite phantom.

The gantry was angled at 270° (Fig. 1(d)) and 500 MUs were delivered for each strip of material covering, including no material for comparison. The 270° gantry configuration gives a dose maximum at depth ($d_{\text{max}}$) of approximately 0.8 cm, according to our commissioning Pdd data. To account for the electron equilibrium effect of ion chamber measurements, amounts of solid water buildup were added in front of the ion chamber for the depth to pass the $d_{\text{max}}$ until the percent dose change from clothing to no clothing was negative.

### C. Evaluate air gap effect

In a clinical situation, it is highly likely to have air gaps between the skin and the gown. In this study, we used a full‐sized cloth and paper gown draped onto the Lucite phantom embedded with the Markus chamber. Two rigid plastic rulers were attached to the top corners of the Lucite block such that the gown would hang at approximately the distance as indicated by the rulers. We took measurements with the gown at the surface, but fitting loosely, so the initial air gap was about 0 cm and then moved to 3 and 5 cm with the gantry angled at 248° and 292° for a combined reading.

### D. Effective water equivalency of cloth gowns

To further understand the dose perturbation by the gown material, the water equivalency of the cloth per layer was evaluated by taking readings from a Farmer chamber (PTW, Freiburg, Germany) using a solid water phantom. We recorded the number of cloth layers that would take to match the readings with a thickness of solid water in place as attenuator. This was done for two solid water thicknesses of 2 mm and 5 mm. The measurements were taken at 100 cm SSD with gantry angle at 0°, in high‐dose rate total skin electron mode with the Lucite spoiler in place.

## III. RESULTS & DISCUSSION

**Figure 2:**
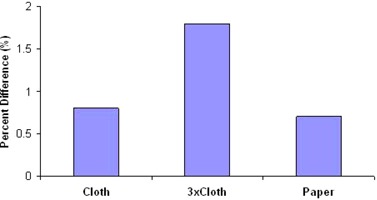
Surface film results showing percent difference of measurements of with gown material relative to ‘no gown'.

For GAFCHROMIC EBT2 films surface dose results, we found the percent differences to be a 0.8% increase in dose for cloth, 1.8% for tri‐layered cloth, and 0.7% for paper (Fig. 2).

**Figure 3:**
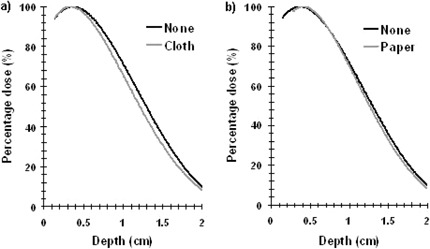
Percentage depth dose (Pdd) profile results showing Pdd obtained with: (a) cloth gown; (b) paper gown.

For the films that were sandwiched within the phantom, there was a small depth shift when comparing the average perturbation of the Pdd profile with and without cloth covering (Fig. 3(a)). For paper gowns, it also showed similar results (Fig. 3(b)).

Markus chamber results at 270° gantry and 0° phantom orientation show a minimal change (<0.2%) in surface dose for both single layer cloth and the paper gown. However, the tri‐layer cloth caused an increase of skin surface dose by 1.4% compared to the no gown scenario. At the depth of 8 mm of solid water, the differences between with and without gown were found to be between −0.2% and −0.4% for all three materials.

**Figure 4:**
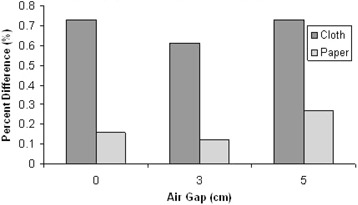
Air gap comparison between cloth and paper at 0° phantom orientation.

Figure 4 shows the Markus ion chamber results with cloth and paper gowns with adjusted air gaps at 248° and 292° gantry angles and 0° phantom orientation. The figure shows that introducing an air gap does not introduce significant dose perturbation.

For the water equivalency of the cloth gowns (Table 1), we found that 11 layers of cloth (∼1cm in thickness for each layer) were needed to equal the ion chamber reading with 2 mm of solid water, whereas 25 layers were needed to equal the reading from 5 mm of solid water.

Table 1 shows that 0.2 mm of solid water is approximately equivalent to 1 layer of cloth. The 0.2 mm depth change is insignificant and it should only introduce a minimal dose variation at $d_{\text{max}}$, as seen in previous results.

**Table 1:** Effective water‐equivalency results of cloth gowns based on ion chamber readings

*Solid Water (mm)*	*Solid Water (nC)*	*Cloth Layers*	*Cloth (nC)*	*% Diff*
2	42.42	11	42.45	0.07
5	34.37	25	34.40	0.09

## IV. CONCLUSIONS

With multiple measuring methods, results of this study show single‐layer paper or cloth hospital gowns do not appear to modulate dose more than 1%. With the addition of an air gap, this did not significantly change dose. However, folds in patient gowns could contribute to greater dose perturbation.

We have shown the dosimetric effect is not substantial for cloth gowns and even less so for paper gowns. Although treating these patients without covering has been standard practice, it is possible that if a patient adamantly refused to disrobe, they may still receive treatment. On the other hand, this dosimetric analysis calls into question the need to treat total skin electron patients completely uncovered. Clinical measurements of skin dose with film during treatments with and without covering may be a reasonable next step in investigating this topic.
